# Design, implementation, and evaluation of a peer-led virtual research training program for early-career health researchers in Central Africa

**DOI:** 10.21203/rs.3.rs-10235482/v1

**Published:** 2026-08-10

**Authors:** Peter Vanes Ebasone, Gabriel Tchatchouang Mabou, Blaise Barche, Clenise Ngwa, Antoinette Tendoh, Jordanne Ching, Melpsa Johney, Kenneth Emeka Enwerem, Suaibu Umaru, Marc Lionel Ngamani, Emmanuel Gwan, Sonita T Abeh, Edouard Faustin Belinga, Donald R. Hoover, Qiuhu Shi, Denis Nash, Kathryn Anastos, André Pascal Kengne, Marcel Yotebieng, Anastase Dzudie

**Affiliations:** Clinical Research Education, Networking and Consultancy (CRENC); Clinical Research Education, Networking and Consultancy (CRENC); Clinical Research Education, Networking and Consultancy (CRENC); Clinical Research Education, Networking and Consultancy (CRENC); Clinical Research Education, Networking and Consultancy (CRENC); Clinical Research Education, Networking and Consultancy (CRENC); Clinical Research Education, Networking and Consultancy (CRENC); Institute of Human Virology Nigeria (IHVN); Clinical Research Education, Networking and Consultancy (CRENC); Clinical Research Education, Networking and Consultancy (CRENC); Clinical Research Education, Networking and Consultancy (CRENC); Clinical Research Education, Networking and Consultancy (CRENC); Clinical Research Education, Networking and Consultancy (CRENC); Department of Statistics and Institute for Health, Health Care Policy and Aging Research, Rutgers, The State University of New Jersey; Department of Public Health, New York Medical College; Institute for Implementation Science in Population Health, Graduate School of Public Health and Health Policy, City University of New York; Department of Medicine, Albert Einstein College of Medicine; Clinical Research Education, Networking and Consultancy (CRENC); Department of Medicine, Albert Einstein College of Medicine; Clinical Research Education, Networking and Consultancy (CRENC)

**Keywords:** research capacity building, peer-led training, virtual learning, sub-Saharan Africa, CRENC-IeDEA, self-efficacy

## Abstract

**Background:**

Sub-Saharan Africa bears a disproportionate share of the global disease burden yet contributes less than 4% of global scientific publications. Most research capacity-building efforts are dominated by short-term workshops that hardly translate into sustained impact. We describe the design, implementation, and evaluation of the CRENC–IeDEA Monday Research Calls, a peer-led, longitudinal virtual research training program for early-career health researchers in Central Africa.

**Methods:**

The program delivered 10 modules across 46 weekly one-hour sessions via Zoom during 2025. Early-career researchers delivered presentations and responded to audience questions under structured senior faculty mentorship. An end-of-program survey (N = 42) assessed satisfaction, self-reported research self-efficacy across 14 competency domains, skill application, and research outputs using the Kirkpatrick evaluation framework. Wilcoxon signed-rank tests with Holm–Bonferroni correction and Cohen's d effect sizes were used for pre–post comparisons.

**Results:**

The program received 227 applications, with 55 participants selected for synchronous training. Respondents were predominantly aged 25–34 years (62%), male (55%), and from Cameroon (83%). Thirty-six percent attended more than 75% of sessions, and 90% would definitely recommend the program. Overall satisfaction was high (mean 4.43/5; Cronbach’s alpha = 0.82), with content applicability rated 4.60/5. Composite self-efficacy increased significantly from a pre-program mean of 3.10 to 4.20 (mean change = 1.10; *p* < 0.001), with large effect sizes across all 14 domains (Cohen’s *d* range: 1.02–1.48). The largest gains were in critical appraisal of literature (*d* = 1.48), conducting literature reviews (*d* = 1.46), and implementation science concepts (*d* = 1.41). Eighty-three percent reported at least one tangible research output, including research proposal development (38%), manuscript contributions (31%), and peer-reviewed publications (14%).

**Conclusions:**

A peer-led, longitudinal virtual training program grounded in adult learning theory and community of practice principles may achieve significant medium-term competency gains and tangible research outputs among early-career researchers in resource-limited settings at modest cost. Practical exercises, bilingual delivery, and prospective evaluation with a comparison group are critical next steps.

## Background

Sub-Saharan Africa (SSA) bears a disproportionate share of the global disease burden yet contributes less than 4% of the world's scientific publications [1-3]. This "10/90" gap reflects not only insufficient funding and infrastructure [4, 5] but a more fundamental deficit: the absence of sustained, contextually embedded training that enables early-career researchers to move from exposure to research concepts toward active scientific participation [2, 5, 6].

Existing capacity-building efforts are dominated by short-term workshops that produce measurable immediate gains in knowledge and confidence but rarely translate into sustained research productivity [1, 7]. Systematic reviews consistently identify the same structural limitations: no longitudinal follow-up, insufficient mentorship, and insufficient opportunity for hands-on practice within an ongoing research community [1, 7]. A longitudinal, low-cost, institutionally embedded model grounded in a coherent pedagogic framework does not yet have a strong evidence base in the African context.

The Clinical Research Education, Networking and Consultancy (CRENC), a Cameroonian not-for-profit research organization, and the Central Africa consortium of the International epidemiology Databases to Evaluate AIDS (CA-IeDEA), an NIH-funded consortium operating across 24 sites in five countries (Burundi, Cameroon, Democratic Republic of Congo, Republic of Congo, and Rwanda), share a mandate to strengthen research capacity among affiliated investigators [6, 8]. Both organizations have previously delivered joint capacity-building activities, including scientific writing workshops [6] and investigator mentorship through the Fogarty–IeDEA mentoring program (FIMP). Despite this established infrastructure, persistent gaps in research skills and productivity among early-career investigators motivated the development of a structured, recurring virtual training program: the CRENC–IeDEA Monday Research Calls, launched in 2022.

Two theoretical frameworks informed the program's design. Knowles' andragogical model holds that adults engage most effectively when content is relevant to their professional context, builds on prior experience, and involves active participation [9]. Wenger's Community of Practice (CoP) framework complements this, emphasising that sustained professional learning occurs through shared practice and social interaction rather than isolated instructional episodes [10]. Peer-led models operationalize both: they situate learning in contextually familiar relationships, build horizontal networks, and maintain scientific rigor when combined with expert oversight [9-11].

This paper describes the design, implementation, and evaluation of the 2025 CRENC–IeDEA program, our most comprehensively evaluated cohort to date. We report participant reach, satisfaction, competency gains, and research outputs, providing evidence for a scalable peer-led virtual model of research capacity building in low- and middle-income countries.

## Methods

### 2.1 Program description

#### Program overview and target audience

Established in 2022 as an internal training initiative for CRENC and CA-IeDEA affiliated staff and students, the program subsequently evolved into an open-access platform for early-career researchers across Central Africa and beyond. The 2025 edition ran from January 20th to December 22nd, with 49 weekly one-hour sessions scheduled on Monday afternoons via Zoom; 46 sessions were delivered (three were cancelled due to scheduling conflicts). A formal call for applications was disseminated through institutional email lists and social media (LinkedIn, WhatsApp, Facebook and Twitter/X). From 227 applicants, 55 were selected for synchronous live participation based on early-career profile, confirmed availability, reliable internet access, and commitment to a minimum attendance requirement; the remaining 173 were enrolled in an asynchronous track with access to session recordings and materials through the program's online learning management system (LearnDash, learn.crenc.org). A certificate of completion was awarded to synchronous participants who attended at least 50% of sessions.

#### Curriculum

The curriculum comprised 10 content modules spanning the full health research cycle, from foundational skills in research question formulation, literature synthesis, research ethics, and study design through to advanced topics in bias and confounding, data analysis, data management, and scientific writing (Table 1). Modules were sequenced to reflect Bloom's taxonomy of cognitive skill development [7], progressing from knowledge acquisition in early modules through analytical and applied competencies in the middle modules, to synthesis and communication in the final modules. Each module included structured learning objectives tailored to early-career researchers, reviewed and approved by consensus among the senior faculty team prior to implementation.

#### Peer-led delivery model and instructional team

Consistent with adult learning theory and Community of Practice principles [9-11], the program adopted a peer-led delivery model in which early-career researchers served as primary session presenters. Each presenter prepared session content based on structured module objectives and shared slides with the senior faculty team at least two weeks before delivery for feedback and quality assurance. Senior faculty participated across all sessions as discussants, providing expert commentary and contextualizing content within broader research practice. In the 2025 edition, 19 individuals served as presenters (9 male, 10 female; three first-time presenters), supported by two program coordinators, a communications lead, and an attendance tracker.

#### Technology and resource requirements

Program delivery relied on accessible, low-cost digital tools to ensure replicability in resource-limited settings. The only additional financial expenditure was a Zoom subscription of approximately US$20 per month. Faculty and program staff contributed their time through their institutional roles or on a voluntary basis, while all other platforms operated through free tiers or existing institutional subscriptions. Session recordings were processed and hosted on the learning management system (LMS), serving as the content backbone for both synchronous and asynchronous participants. The full technology stack is summarized in Table 2.

### 2.2 Evaluation design and analysis

#### Study design and data sources

This was a descriptive program evaluation using a single-group pre–post survey design guided by the Kirkpatrick Evaluation Framework [12]. Data were drawn from three complementary sources: (1) an end-of-program survey administered via Microsoft Forms to all synchronous participants at program completion; (2) weekly session attendance records maintained in Microsoft Excel; and (3) a program documentation template completed by the coordination team across the program year.

#### Survey instrument and Kirkpatrick framework

The survey assessed four domains corresponding to the Kirkpatrick evaluation levels. At Level 1 (Reaction), participants rated satisfaction with program delivery, content, materials, mentorship, organisation, and pace, and indicated likelihood of recommending the program. At Level 2 (Learning), research self-efficacy across 14 domains was assessed before and after the program using a 5-point Likert scale (1 = very low to 5 = very high confidence), with open-ended items capturing least-valued aspects and suggestions for improvement. At Level 3 (Behaviour), participants reported research activities undertaken since joining the program, professional connections formed, and concrete plans to apply learning. At Level 4 (Results), participants reported tangible research outputs including proposals, ethics applications, manuscripts, conference presentations, and funding received. The survey also collected participant demographics and prior research experience; the full framework application is summarised in Supplementary Table S1.

#### Statistical and qualitative analysis

Descriptive statistics, including frequencies, percentages, means, standard deviations, and medians as appropriate, summarized participant characteristics, attendance, and satisfaction. Pre–post self-efficacy comparisons used Wilcoxon signed-rank tests with Holm–Bonferroni correction across all 14 domains; effect sizes were estimated using Cohen's d. Thematic analysis was applied inductively to open-ended responses, with themes coded independently by two team members and reconciled by consensus. Internal consistency of composite scales was assessed using Cronbach's alpha. All quantitative analyses were conducted in R version 4.5.1 (2025-06-13)

This study analyzed anonymized data collected through a routine program evaluation. Participation in the survey was voluntary, and all respondents provided informed consent for the use of their anonymized responses for research and publication purposes.

## Results

### Program reach and participation

The 2025 programme received 227 applications; 55 were selected for synchronous live participation and 173 were enrolled in the asynchronous track (Fig. 1). Among the 55 synchronous participants, mean attendance across 46 delivered sessions was 60.8% (SD 24.4%; median 63%). Three participants achieved 100% attendance, 36 (65%) met the certificate eligibility threshold of ≥ 50% of sessions, and 36% attended more than 75% of sessions. Attendance was highest in the early months and declined gradually, from a mean of 67% per session in the first quarter to 54% in the final quarter (Supplementary Figure S1). Forty-two of the 55 synchronous participants completed the end-of-program survey (response rate: 76%).

### Participant characteristics

Respondents were predominantly aged 25–34 years (n = 26, 62%), mostly from Cameroon (n = 35, 83%), with smaller representation from Rwanda (n = 6, 14%) and the Democratic Republic of Congo (n = 1, 2.4%), and quite evenly split by gender (male: n = 23, 55%; female: n = 19, 45%). Institutional affiliations spanned universities, research institutes, hospitals, and NGOs or government bodies. Most had limited to moderate prior research experience: 4 (10%) had none, 15 (36%) had some exposure through coursework or project assistance, 16 (38%) had completed one to two independent projects, and 7 (17%) had substantial experience. Full characteristics are presented in Table 3.

### Satisfaction and program experience (Kirkpatrick Level 1: Reaction)

Overall program satisfaction was high, with a composite mean score of 4.43 out of 5 (SD 0.70; Cronbach's alpha = 0.82). Mean ratings across satisfaction items ranged from 4.29 for knowledge gained to 4.60 for content applicability and usefulness of materials, with all items rated above 4.0 (Fig. 2). Eighty-six percent of respondents rated program difficulty and pace as appropriate, and 38 of 42 (90%) indicated they would recommend the program to colleagues. Module relevance and teaching quality ratings were consistent across all 10 modules (Supplementary Table S2). When asked to nominate the most valuable module, participants most frequently selected Module 1 (Research Questions, Aims and Hypotheses; Supplementary Figure S2).

Thematic analysis of open-ended feedback on most valued aspects (n = 33) identified five themes: mentorship and expert feedback from senior faculty (n = 10), quality and accessibility of presentation materials (n = 8), interactive Q&A and real-time faculty commentary (n = 8), program consistency and organization (n = 7), and relevance to professional development (n = 4). The integration of peer-led delivery with senior oversight was highlighted as a defining program strength *("The professionalism, timeliness, consistency and mentorship from the Profs and experts"*). Conversely, feedback on least valued aspects (n = 26) identified four areas for improvement: insufficient hands-on practice with data analysis tools (n = 8), scheduling conflicts and perceived time constraints in technical modules (n = 5), limited participant interaction beyond faculty-led Q&A and absence of modular assignments (n = 4), and exclusively English-language delivery limiting francophone engagement (n = 3); three respondents reported no concerns. Participant suggestions (n = 27) directly mirrored these gaps, prioritizing practical exercises with real datasets, bilingual delivery, and structured assessments to consolidate learning.

### Self-reported research self-efficacy gains (Kirkpatrick Level 2: Learning)

Self-reported research self-efficacy increased significantly across all 14 competency domains, with the composite score rising from a pre-program mean of 3.10 to 4.20 (mean change = 1.10, SD = 0.66; Wilcoxon V = 779, p < 0.001; pre-programme Cronbach's alpha = 0.95; post-programme alpha = 0.93). All domains showed large, statistically significant improvements after Holm–Bonferroni correction (Cohen's d range: 1.02–1.48; all p < 0.001; Supplementary Table S3). Participants rated overall knowledge and skill gains at a mean of 4.29/5 (SD 0.67) and applicability to their work at 4.60/5 (SD 0.59). The individual-level shift across all domains is shown in Fig. 3.

Each row represents one respondent. Competency domains: a = formulating research questions; b = conducting literature reviews; c = understanding research ethics and GCP; d = selecting study designs; e = implementation science concepts; f = scientific writing (basic); g = bias, confounding and missing data; h = data analysis and interpretation; i = scientific writing (advanced); j = data collection and management; k = critical appraisal of literature; l = developing a research proposal; m = writing and submitting manuscripts; n = grant writing and research funding. Colour scale: red = 1 (very low confidence); green = 5 (very high confidence); grey = missing response. One respondent with no competency responses was excluded: remaining missing values handled by pairwise deletion (effective N = 39–41 per domain). Full domain-level statistics are provided in Supplementary Table S4. GCP = Good Clinical Practice.

The highest absolute mean change was in formulating research questions (ΔM = 1.34). The largest standardised gains were in critical appraisal of literature (Cohen’s d = 1.48), conducting literature reviews (d = 1.46), and implementation science concepts (d = 1.41), domains participants identified as foundational gaps at entry: *"As a beginner, this course clarified the challenges I had in formulating good research aims and hypothesis."* Large gains were also observed in formulating research questions (d = 1.35) and data collection and management (d = 1.34).

The smallest gains, though still large by conventional thresholds, were in writing and submitting manuscripts (d = 1.02), data analysis and interpretation (d = 1.04), and grant writing (d = 1.16), applied technical domains participants consistently identified as requiring more hands-on practice. In subgroup analyses, no significant differences in self-efficacy change were found by gender or program role, except those participants with prior research experience showed significantly greater gains in the statistical analysis domain (adjusted p = 0.005).

### Skill application and research outputs (Kirkpatrick Levels 3 and 4: Behaviour and Results)

#### Level 3 - Behaviour.

Thirty-five of 42 respondents (83%) reported applying program-acquired skills to at least one research activity. The most common applications were developing a new research question or concept (n = 21, 50%), writing or revising a research proposal (n = 16, 38%), and mentoring or teaching others (n = 16, 38%; Fig. 4). More than half (n = 22, 52%) reported forming meaningful professional connections through the program.

Thematic analysis of 29 open-ended responses identified four themes around planned application of learning. The most prominent was academic progression, translating acquired skills into formal scholarly milestones: *"I plan on completing my PhD proposal and submitting it for funding."* The second was research productivity, with participants describing specific manuscript and publication plans: *"Oui, publication d'articles sur le VIH" [Yes, publication of articles on HIV].* The third was teaching and capacity transfer, extending skills to peers and students: *"I plan to assist students in refining their research questions and developing robust study designs."* The fourth was applied research practice, with clinicians integrating training directly into ongoing work: *"I plan to integrate the training into ongoing depression screening programs by enhancing cohort coordination protocols for mental health research."*

#### Level 4 - Results.

Reported tangible outputs included contributing to or writing a manuscript (n = 13, 31%), submitting an ethics application (n = 9, 21%), receiving ethics approval (n = 9, 21%), starting data collection (n = 9, 21%), presenting at a conference or seminar (n = 7, 17%), submitting a manuscript for publication (n = 7, 17%), completing data analysis (n = 6, 14%), publishing a peer-reviewed paper (n = 6, 14%), and receiving research funding (n = 3, 7%). Among the 35 respondents reporting at least one output, a mean of 3.07 output types was reported. Six respondents reported no outputs at the time of survey completion, and one abstract acceptance was independently confirmed as a verifiable output. The full distribution is shown in Fig. 4.

## Discussion

The CRENC–IeDEA programme evaluation suggests that a peer-led, longitudinal virtual training model can strengthen research competencies and support early scholarly productivity among health researchers in resource-limited settings as evaluated using the Kirkpatrick model. Short-term and workshop-based approaches in the region have consistently shown positive immediate effects on knowledge but limited translation into sustained outputs [1, 2, 12]. The identification of peer-reviewed publications, ethics approvals, and research funding within a single year-long program therefore represents a stronger output profile than such approaches typically report, suggesting that a longitudinal, institutionally embedded virtual model can support meaningful research capacity development where access to sustained training and mentorship remains limited.

These findings are best understood through the program's design logic. Its emphasis on practical relevance, progressive engagement across the research cycle, and active participation reflects core principles of adult learning and helps explain why the program supported not only knowledge uptake but also its application to ongoing research activity [9]. The peer-led format, reinforced by senior faculty discussants, functioned as a community of practice in which learning was socially grounded while academic rigor was maintained [10]. Similar blends of peer and expert mentorship have been described in African research capacity initiatives as strengthening not only individual research competence but also the broader research culture within which early-career investigators develop [13, 14]. The program's value therefore lies not only in being well received, but also in supporting movement from learning about research toward actively participating in it.

The pattern of gains is consistent with the program's theoretical design. Foundational domains such as research question formulation, literature review, and critical appraisal showed the largest improvements, reflecting the structured, progressive curriculum and the relevance of these skills to participants' immediate research needs. Smaller gains in technically demanding domains such as data analysis, manuscript submission, and grant writing reflect a recognised boundary of seminar-based virtual training, where guided practice around real tasks is difficult to replicate [12], and align directly with participants' own calls for more hands-on practical exercises. That more than four-fifths of respondents applied program skills to at least one research activity, and that outputs included peer-reviewed publications, ethics approvals, and funding awards, suggests movement beyond knowledge acquisition toward substantive scholarly activity.

Sustaining synchronous engagement across a full year remains a genuine implementation challenge. Attendance in this program declined modestly across the year, corresponding in part to technically demanding modules and reflecting competing demands of clinical work, postgraduate training, and variable internet connectivity. The asynchronous track served as a deliberate design buffer, allowing participants who missed live sessions to maintain curriculum progress, though future iterations would benefit from complementing plenary sessions with smaller practical workshops or mentored exercises, particularly for technical modules. The language barrier identified by francophone participants adds an equity dimension the program is actively addressing: a dedicated French-language edition is planned to broaden access across a region where French is the dominant professional language [2]. Beyond a Zoom subscription, delivery relied entirely on free or institutionally available platforms, demonstrating that meaningful research training does not require high-cost infrastructure when supported by strong coordination, committed faculty, and a clear pedagogic framework. Scalability, however, must be matched by instructional depth and applied learning opportunities if early gains are to translate into sustained research participation [1].

An important finding of this evaluation is the feasibility of sustaining a longitudinal, peer-led virtual research training program. The program has been established as an annual offering, with each cohort informing curriculum refinements based on participant feedback and evolving training needs. The ongoing 2026 French-language edition demonstrates the model’s adaptability and supports its potential as a sustainable approach to research capacity building in resource-limited settings.

Several limitations warrant consideration. Self-reported outcomes at Levels 2 to 4 are vulnerable to recall and social desirability bias; pre–post self-efficacy was measured retrospectively at a single time point, which may have introduced response shift bias in participants' estimation of their baseline competence. The absence of a comparison group precludes causal attribution, and the end-of-program design does not capture longer-term retention or sustained behavior change. Transferability may be limited in settings without an established research consortium or minimum conditions required such as internet access, faculty availability and organizational structure. Finally, it is possible that our selection method of participants might have led to very motivated applicants, precluding generalizability in other contexts. Against these limitations, the study draws on multiple complementary data sources, applies a recognized evaluation framework across all four Kirkpatrick levels, and combines quantitative and qualitative evidence to capture both what participants gained and how they experienced the program. The program's embedding within an active research consortium further distinguishes it from isolated training initiatives, and the fact that 37 of 42 respondents (88%) consented to follow-up contact provides a basis for prospective outcome assessment. Last but not the least, while female participation in research remains a strategic priority across Africa [15], our program achieved near gender balance among participants (45% female) and presenters (53% female), suggesting that peer-led, accessible virtual models may help reduce structural barriers and promote more equitable participation of women in research capacity-building initiatives.

Future evaluations should incorporate prospective pre–post assessment, objective outcome measures, longer follow-up, and a comparison group, with particular attention to technical domains where confidence gains alone may not be sufficient to support sustained research performance.

## Conclusions

The CRENC–IeDEA research training program demonstrated that a peer-led, longitudinal virtual model grounded in adult learning theory and community of practice principles can achieve meaningful competency gains and tangible research outputs among early-career health researchers in a resource-limited setting, at a cost accessible to similar organizations across the region. Practical exercises, bilingual delivery, and prospective evaluation with a comparison group are the critical next steps for strengthening both the program and the evidence base for scalable research capacity building in low- and middle-income countries.

## Supplementary Material

This is a list of supplementary files associated with this preprint. Click to download.

• SupplementaryTableandfigure.docx

## Figures and Tables

**Figure 1:**
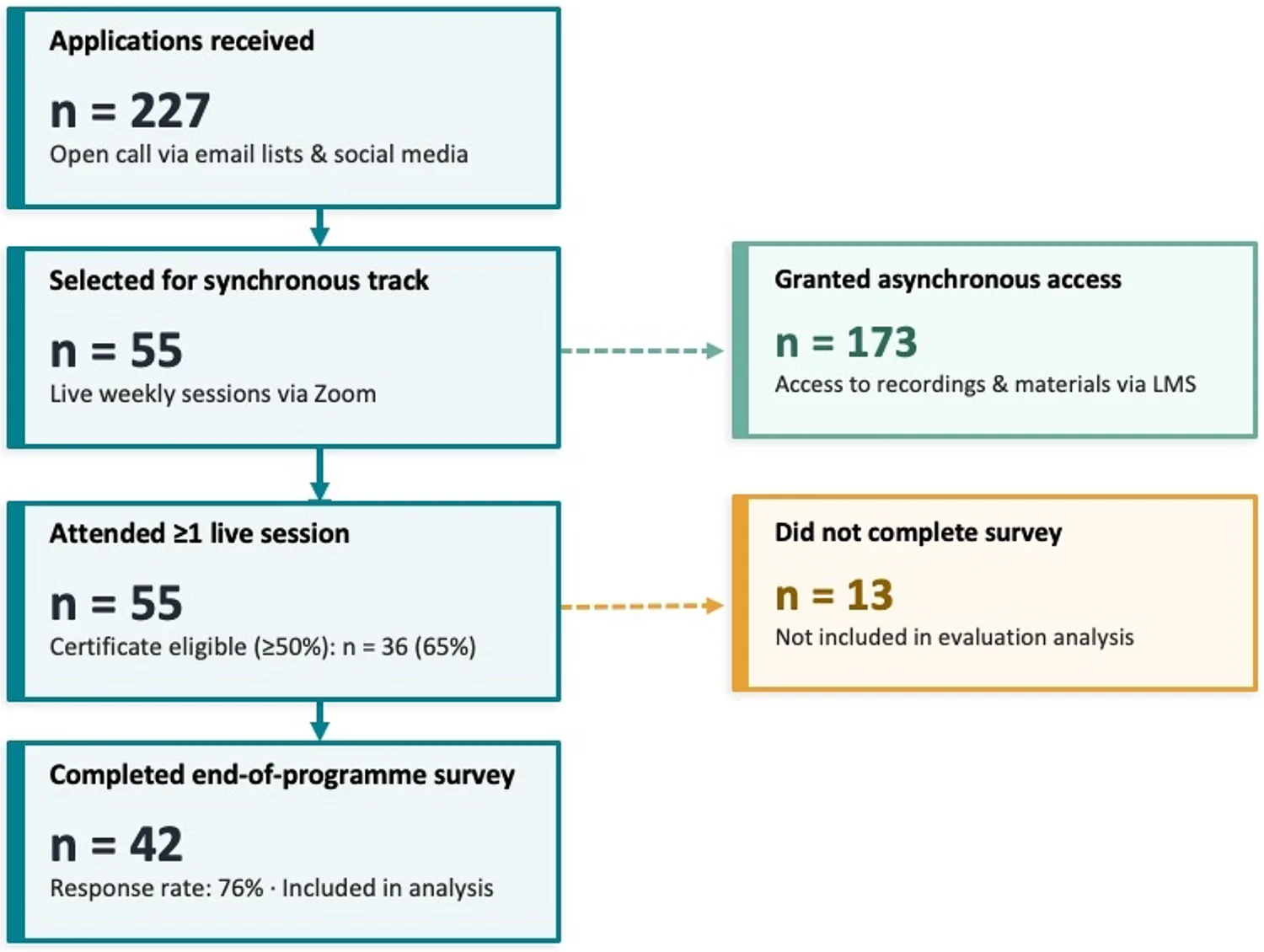
Study Participant Flow for the CRENC–IeDEA Capacity Building Program 2025

**Figure 2:**
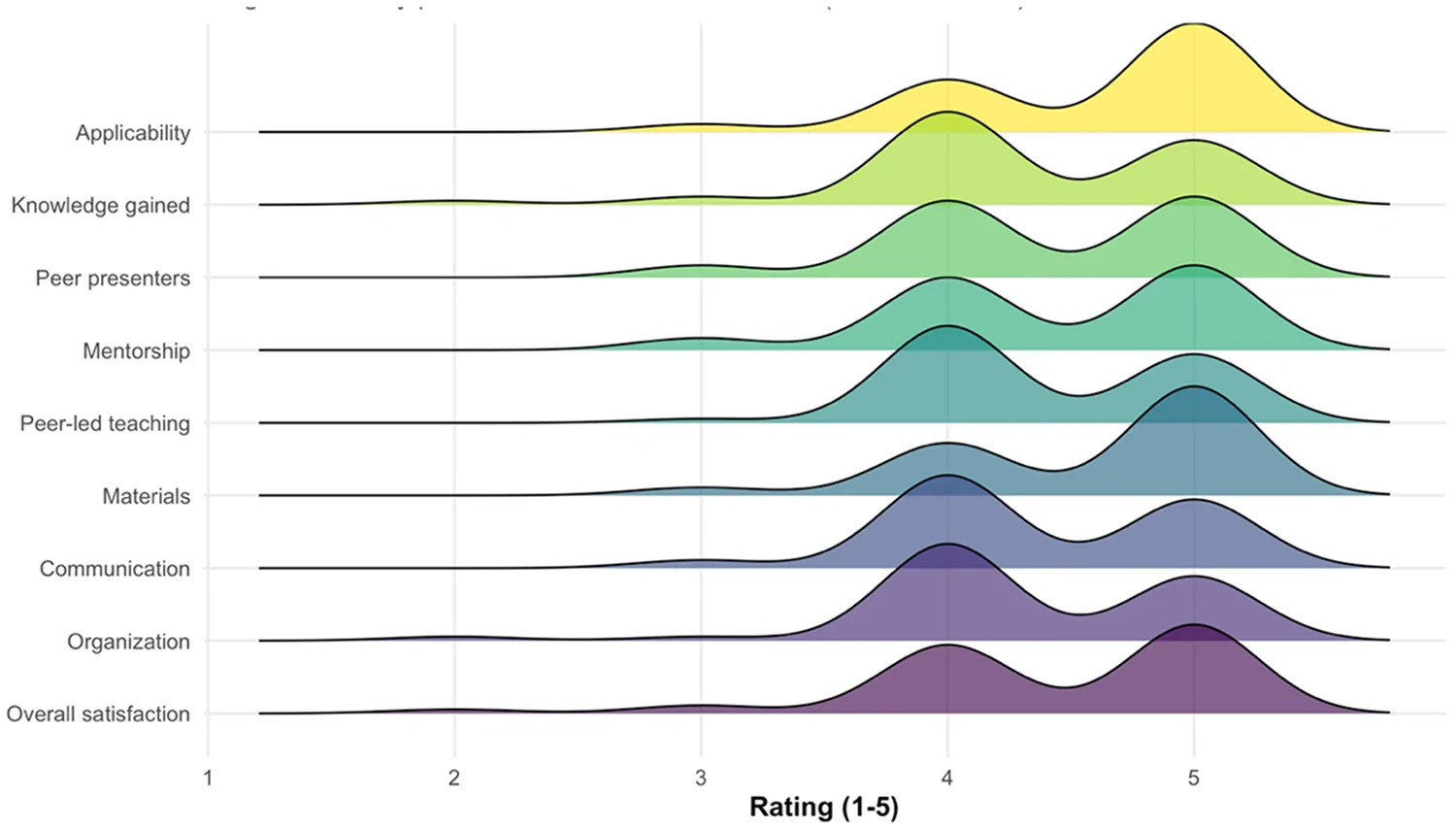
Ridgeplot of the distribution of satisfaction ratings across program evaluation items (N = 42). Ratings were recorded on a 5-point Likert scale (1 = not at all satisfied; 5 = very satisfied).

**Figure 3:**
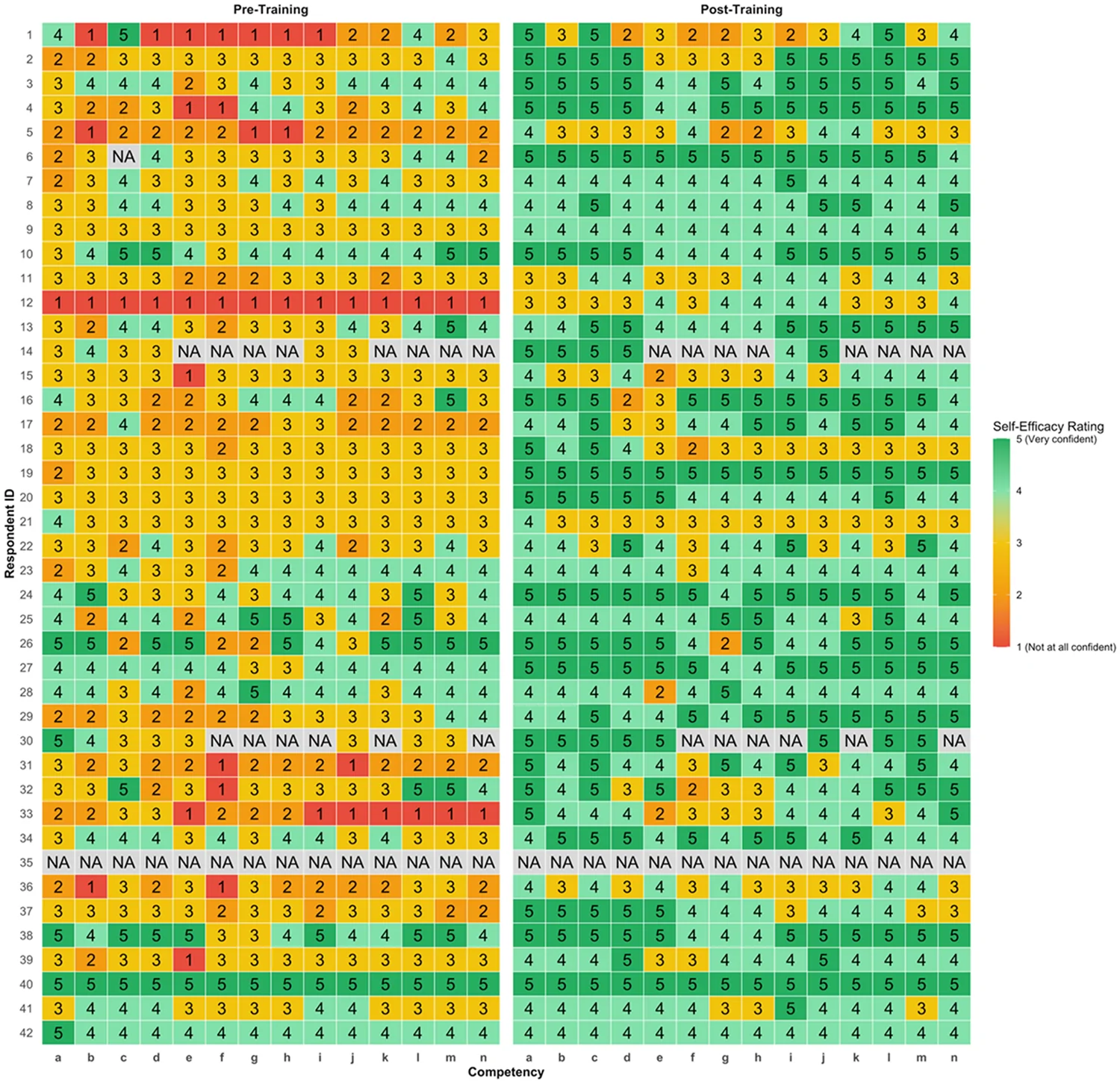
Pre- and post-program self-reported research self-efficacy across 14 competency domains (N = 41). Each row represents one respondent. Competency domains: a = formulating research questions; b = conducting literature reviews; c = understanding research ethics and GCP; d = selecting study designs; e = implementation science concepts; f = scientific writing (basic); g = bias, confounding and missing data; h = data analysis and interpretation; i = scientific writing (advanced); j = data collection and management; k = critical appraisal of literature; l = developing a research proposal; m = writing and submitting manuscripts; n = grant writing and research funding. Colour scale: red = 1 (very low confidence); green = 5 (very high confidence); grey = missing response. One respondent with no competency responses was excluded: remaining missing values handled by pairwise deletion (effective N = 39–41 per domain). Full domain-level statistics are provided in Supplementary Table S4. GCP = Good Clinical Practice.

**Figure 4:**
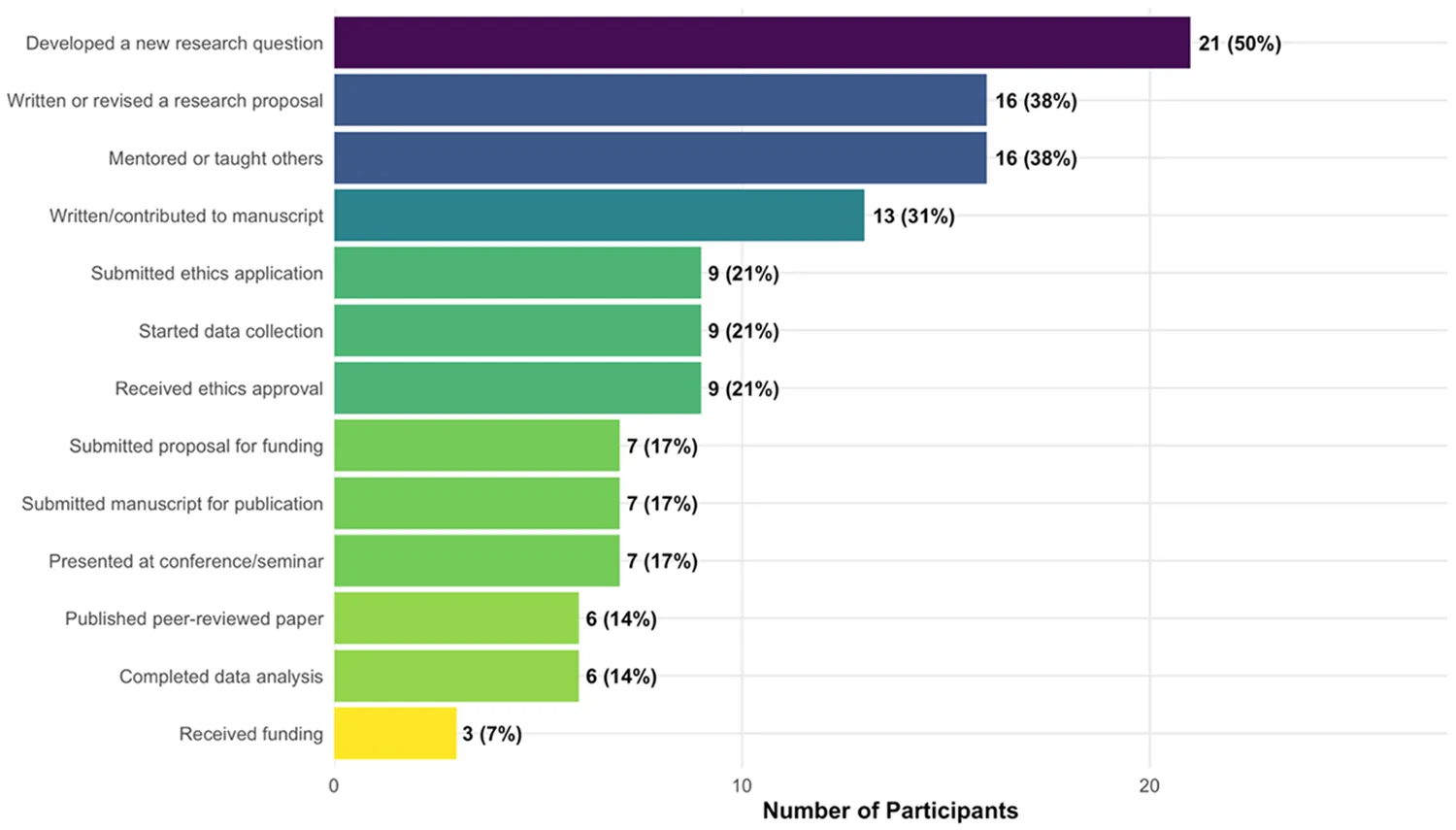
Research outputs reported by survey respondents since joining the program (N = 42). Participants could select multiple output types; percentages are of total N and are not mutually exclusive. All outputs are self-reported unless otherwise indicated.

**Table 1:** CRENC–IeDEA 2025 curriculum: modules, topics, and periods

No.	Module title	Core topics	Period (2025)
1	Research questions, aims and hypotheses	Formulating PICO questions, study aims, null/alternative hypotheses	Jan–Feb
2	Literature review and synthesis	Search strategies, critical appraisal, referencing tools	Feb–Mar
3	Research ethics and Good Clinical Practice (GCP)	Ethical principles, informed consent, IRB processes, GCP certification	Mar–Apr
4	Study designs in medical research	Observational, experimental, and mixed-method designs	Apr–May
5	Implementation science basics	Implementation frameworks, theories, and application to health practice	May–Jun
6	Scientific writing - Part I	Structure of a manuscript, writing the Introduction and Methods	Jun–Jul
7	Bias, confounding, and missing data	Types of bias, confounding control, sensitivity analyses	Jul–Aug
8	Data analysis and interpretation	Descriptive and inferential statistics, software overview, results interpretation	Sep–Oct
9	Scientific writing - Part II	Writing Results, Discussion, and Abstract; responding to peer review	Oct–Nov
10	Data collection and management	Data collection tools, database design, quality control, FAIR principles	Nov–Dec

GCP = Good Clinical Practice. All sessions were peer-led, with senior expert mentors present as discussants across the full program.

**Table 2:** Technology platforms used in the CRENC–IeDEA 2025 program

Function	Tool	Notes
Live session delivery	Zoom	~US$20/month for up to 100 attendees
Learning management system (LMS)	LearnDash (learn.crenc.org)	WordPress-based; hosted module recordings and materials for both synchronous and asynchronous participants
Session recording	Zoom Cloud, Google Drive, YouTube (unlisted)	Recordings downloaded, reviewed, trimmed, then uploaded to YouTube and embedded in LMS
Material sharing	WhatsApp groups, Google Drive, LMS	Slides shared via WhatsApp after each session and uploaded to Google Drive; linked through LMS
Formal communication	HubSpot (free tier)	Stratified mailing lists for synchronous, asynchronous, and faculty groups
Informal communication	WhatsApp	Session reminders and coordination
Attendance tracking	Microsoft Excel	Completed manually during each live session
Survey and feedback	Microsoft Forms, Google Forms	Microsoft Forms for end-of-program survey; Google Forms for call for applications
Certificate generation	PowerPoint, Adobe Photoshop	Designed and issued to qualifying participants

LMS = learning management system. All platforms except Zoom were used at no direct cost through free tiers or existing institutional subscriptions.

**Table 3:** Characteristics of survey respondents (N = 42)

Characteristic	n (%)
Age range	
18–24 years	3 (7.1%)
25–34 years	26 (61.9%)
35–44 years	8 (19.0%)
45–54 years	5 (11.9%)
Gender	
Female	19 (45.2%)
Male	23 (54.8%)
Country of residence	
Cameroon	35 (83.3%)
Rwanda	6 (14.3%)
DR Congo	1 (2.4%)
Institutional affiliation	
University or academic institution	14 (33.3%)
Research institute	11 (26.2%)
Hospital or clinical setting	8 (19.0%)
NGO, government, or other	9 (21.4%)
Prior research experience	
No prior experience	4 (9.5%)
Some exposure (coursework or assisted on projects)	15 (35.7%)
Moderate experience (1–2 completed projects)	16 (38.1%)
Substantial experience (3 + projects or publications)	7 (16.7%)

## Data Availability

De-identified survey data and the survey instrument are available from the corresponding author upon reasonable request.
